# Survival from cancer of the colon in England and Wales up to 2001

**DOI:** 10.1038/sj.bjc.6604578

**Published:** 2008-09-23

**Authors:** E Mitry, B Rachet, M J Quinn, N Cooper, M P Coleman

**Affiliations:** 1 Département d'Hépatogastroentérologie et Oncologie Digestive, Centre Hospitalo-Universitaire Ambroise-Paré, 9 avenue Charles de Gaulle, Boulogne F-92100, France; 2 Cancer Research UK Cancer Survival Group, Non-Communicable Disease Epidemiology Unit, Department of Epidemiology and Population Health, London School of Hygiene and Tropical Medicine, Keppel Street, London WC1E 7HT, UK; 3 Social and Health Analysis and Reporting Division, Office for National Statistics (Room FG/114), 1 Myddelton Street, London EC1R 1UW, UK

Colon cancer is one of the most common malignancies in adults worldwide (Parkin, 2001). About 17 000 new cases are diagnosed each year in England and Wales, where it is the second and third most frequent cancer in women and men, respectively. Over the last 25 years, incidence has risen slightly in men, but has remained fairly stable in women (Quinn *et al*, 2001). Of the improvements in survival reported in Europe during the 1990s, one of the largest absolute increases was for large bowel cancer (colon and rectum combined). Survival increased more slowly in the UK during this period than in other western European countries (Sant *et al*, 2001). Substantial deprivation gradients in colon cancer survival have been reported, both in England and Wales (Coleman *et al*, 1999) and in other countries (Auvinen, 1992; Monnet *et al*, 1993). These have been attributed in part to differential access to chemotherapy (Corazziari *et al*, 2004) or specialised treatment centres (Herbert *et al*, 2002; Satagopan *et al*, 2004).

We analysed the data for 206 879 adults diagnosed with colon cancer in England and Wales during the period 1986–1999, some 83% of the 250 445 eligible. About 12% of patients who were otherwise eligible for analysis were excluded with zero recorded survival (date of diagnosis same as date of death). Up to a third of these may in fact have died on the day of diagnosis (Coleman *et al*, 1999), but most were registered solely from a death certificate, and the two categories could not be distinguished in national data. For these two groups of patients, survival was either actually zero or shorter than average (Berrino *et al*, 1995), respectively, and their exclusion leads to some inflation of the survival estimates. The proportion of cases with zero recorded survival was similar in all socioeconomic groups, however, and it declined steadily during the 1990s, again similarly in all socioeconomic groups (data not shown). Exclusions from analysis for zero survival are thus unlikely to have had any substantial impact on socioeconomic gradients in survival, or on trends in the gradient. A further 3.5% of patients with colon cancer were excluded because it was not their first primary cancer.

The proportion of colon tumours recorded as adenocarcinoma rose from 60 to 72% by 1999, but the proportion of poorly specified carcinomas fell from 31 to 14%, suggesting improvement in the quality of pathology data, and that the true proportion of adenocarcinomas was probably approximately 70% throughout the 1990s. A very similar pattern was seen in the pathology of rectal cancers. Information on the clinical stage at diagnosis was not generally available in the national cancer registry before 1995, and stage-specific analyses for the period 1986–1999 were thus not possible.

Annual incidence rates in both men and women rose by about one-fifth overall during 1986–1999, but the increase was notably smaller in the most deprived group, also in both sexes (Figure 1).

## Survival trends

**Figure 2:**
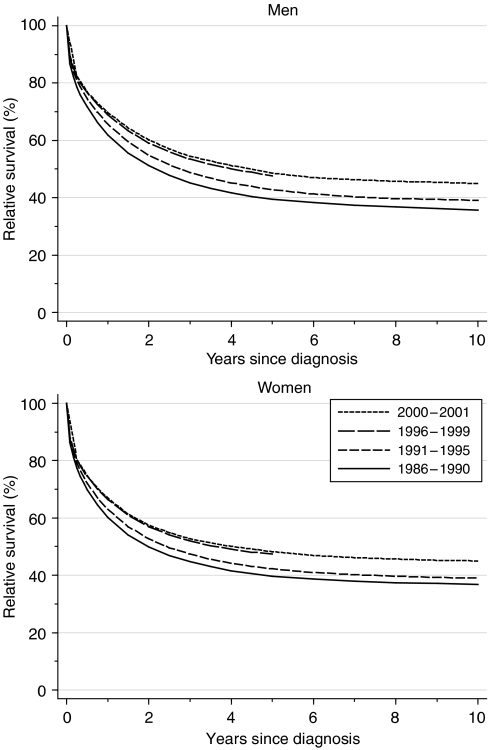
Relative survival (%) up to 10 years after diagnosis by sex and calendar period of diagnosis: England and Wales, adults (15–99 years) diagnosed during 1986–1999 and followed up to 2001. Survival estimated with cohort or complete approach (1986–1990, 1991–1995, 1996–1999) or hybrid approach (2000–2001) (see Rachet *et al*, 2008).

Relative survival up to 10 years after diagnosis has increased substantially in both sexes (Figure 2). In men, 1-year survival rose from 61.9% for those diagnosed in the late 1980s to 68.9% for those diagnosed in the late 1990s. This represents a significant average increase of 5.5% every 5 years, after adjustment for deprivation. Five-year survival rose from 39.5 to 47.6% (+5.6% every 5 years) over the same period. The survival rates and trends over time for women were closely similar to those for men (Table 1).

Short-term predictions of survival for patients diagnosed during 2000–2001, using hybrid analysis (Brenner and Rachet, 2004), suggest that the rapidly increasing trends in survival up to 10 years after diagnosis observed during the 1990s are likely to continue in the near future (Table 1).

## Deprivation

**Figure 3:**
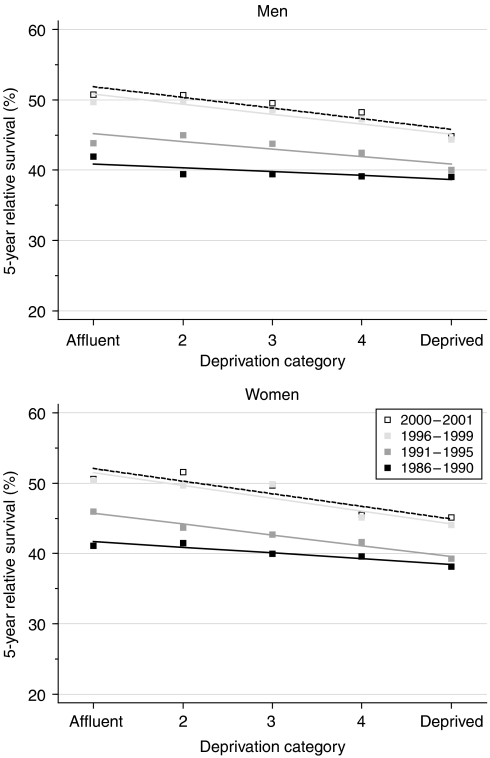
Trends in the deprivation gap in 5-year relative survival (%) by sex and calendar period of diagnosis: England and Wales, adults (15–99 years) diagnosed during 1986–1999 and followed up to 2001.

Survival has been significantly lower for both men and women in the most deprived socioeconomic groups of the population since 1986 (Figure 3). The deprivation gap in 1-year survival has been similar for men and women, and it has widened steadily and significantly in both sexes, reaching −7% for patients diagnosed during 1996–1999 (Table 2). For 5-year survival, the survival deficit for the poorest patients has been slightly but consistently wider for women than men. It also widened steadily and significantly from 1986 to 2001. Between 1986–1990 and 1996–1999, the deprivation gap in 5-year survival increased from −2.2 to −5.7% in men (fitted average change −1.9% every 5 years) and from −3.3 to −7.3% in women (−2.2% every 5 years).

Short-term predictions of survival by socioeconomic group, again using hybrid analysis, do not suggest any imminent reduction in the deprivation gap in survival up to 10 years (Table 2).

## Comment

Substantial improvements in colon cancer survival have occurred for patients who were diagnosed in England and Wales during the 14-year period 1986–1999 and followed up to the end of 2001. The increase in survival for patients diagnosed during the 1990s has been more rapid than for those diagnosed in the 1970s or 1980s (Coleman *et al*, 1999). These trends are coherent with the continuing decline in mortality and the steady rise in incidence over the same period (Quinn *et al*, 2001). The increase in survival may be attributable to improvements in surgery (McArdle and Hole, 2002) and to reduction in operative mortality (Mitry *et al*, 2002). Taken together, the trends suggest that national improvements in colon cancer survival may be attributable both to earlier diagnosis and to improved treatment.

It seems unlikely that the divergence in incidence trends between the poorest fifth of the population and all four of the more affluent groups could be attributable to earlier diagnosis, with colonoscopy somehow being less accessible to the least affluent fifth of society alone, in part because the divergence in incidence has persisted for at least 10 years. Nor could such a pattern plausibly explain the widening deprivation gap in survival, as benign and *in situ* tumours were all excluded from analysis: only first primary, invasive malignant tumours were included. Bias in both the overall survival estimates and the socioeconomic gap in survival could, in principle, arise from the exclusion of a substantial minority of patients whose recorded survival was zero, and for many of whom the actual duration of survival was unknown. This cannot plausibly explain the increasing deprivation gap in survival, however, as the proportion of patients with zero recorded survival was closely similar in all deprivation groups, throughout the 1990s.

Improvements in colon cancer survival in the 15 years up to 2001 were more marked among residents of wealthier districts, both men and women. As a result, the deprivation gap in survival between the most affluent and the most deprived groups increased steadily. The relative survival estimates take account of the divergent trends in overall mortality between socioeconomic groups over the 15 years up to 2001. This suggests that more affluent patients have benefited preferentially from progress in early diagnostic procedures and in access to optimal treatment over this period.

Other evidence also suggests that the deprivation gradient in relative survival for patients diagnosed in England and Wales during the 1990s is likely to be due to a combination of earlier diagnosis and better treatment in more affluent groups. Colorectal cancer patients living in more deprived areas who were diagnosed in southeast England during 1992–1995 were more likely to be admitted as an emergency and less likely to have surgery (Pollock and Vickers, 1998). Adjustment for stage at diagnosis did not abolish the deprivation gradient in survival seen in this region (Schrijvers *et al*, 1995). Population-based colorectal cancer survival also varies with socio-economic status in the United States (Singh *et al*, 2004), but patients from different racial or socioeconomic groups who receive the same treatment appear to have similar outcomes, whether this is in equal access health systems such as the US Veterans Administration (Rabeneck *et al*, 2003), or within clinical trials (Dignam *et al*, 1999). Data from France (Mitry *et al*, 2005) suggest that adjuvant chemotherapy after resection with curative intent could account for much of the national improvement in colon cancer survival in England and Wales during the 1990s, and the increasing deprivation gradient in survival may therefore reflect better access to optimal treatment for patients living in more affluent areas.

## Figures and Tables

**Figure 1:**
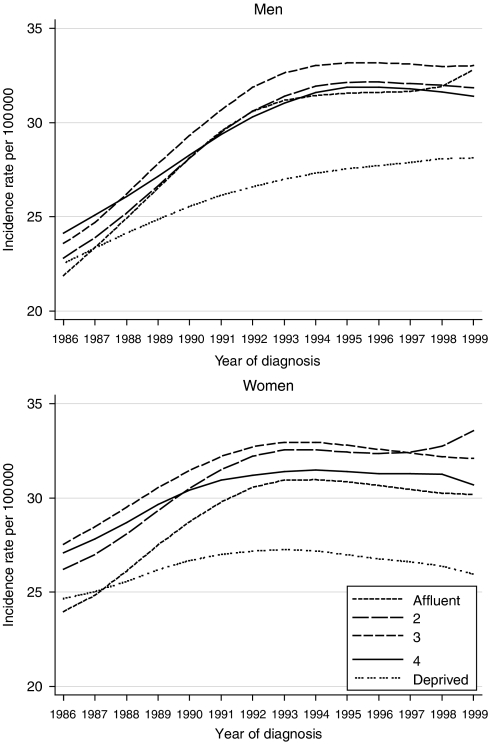
Trends in the age-standardised incidence of colon cancer in adults aged 15–99 years, by sex and deprivation group: England and Wales, 1986–1999.

**Table 1:** Trends in relative survival (%) by sex, time since diagnosis and calendar period of diagnosis: England and Wales, adults (15–99 years) diagnosed during 1986–1999 and followed up to 2001

		**Calendar period of diagnosis^a^**						**Average change (%)**		**Prediction^c^ for patients**	
		**1986–1990**		**1991–1995**		**1996–1999**		**every 5 years^b^**		**diagnosed during 2000–2001**	
**Time since diagnosis**		**Survival (%)**	**95% CI**	**Survival (%)**	**95% CI**	**Survival (%)**	**95% CI**	**Survival (%)**	**95% CI**	**Survival (%)**	**95% CI**
1 year	Men	**61.9**	(61.3, 62.5)	**65.6**	(65.1, 66.2)	**68.9**	(68.4, 69.5)	5.5^**^	(4.5, 6.6)	**69.7**	(68.9, 70.4)
	Women	**60.1**	(59.6, 60.7)	**62.9**	(62.4, 63.4)	**66.5**	(65.9, 67.0)	4.4^**^	(3.3, 5.4)	**66.9**	(66.1, 67.7)
5 years	Men	**39.5**	(38.9, 40.2)	**42.8**	(42.2, 43.4)	**47.6**	(46.8, 48.3)	5.6^**^	(4.2, 7.0)	**48.6**	(47.6, 49.5)
	Women	**39.7**	(39.1, 40.3)	**42.2**	(41.7, 42.8)	**47.4**	(46.7, 48.1)	5.6^**^	(4.3, 6.8)	**48.2**	(47.3, 49.1)
10 years	Men	**35.7**	(35.0, 36.5)	**39.0**	(38.2, 39.8)			5.2^**^	(2.5, 7.9)	**44.9**	(43.8, 46.0)
	Women	**36.8**	(36.2, 37.5)	**39.0**	(38.4, 39.7)			5.3^**^	(2.9, 7.7)	**45.0**	(44.0, 46.0)

CI=confidence interval.

a Survival estimated with cohort or complete approach (see Rachet *et al*, 2008).

b Mean absolute change (%) in survival every 5 years, adjusted for deprivation (see Rachet *et al*, 2008).

c Survival estimated with hybrid approach (see Rachet *et al*, 2008).

^**^P<0.01.

**Table 2:** Trends in the deprivation gap in relative survival (%) by sex, time since diagnosis and calendar period of diagnosis: England and Wales, adults (15–99 years) diagnosed during 1986–1999 and followed up to 2001

		**Calendar period of diagnosis^a^**						**Average change (%)**		**Prediction^c^ for patients**	
		**1986–1990**		**1991–1995**		**1996–1999**		**every** **5 years^b^**		**diagnosed during 2000–2001**	
**Time since diagnosis**		**Deprivation gap (%)**	**95% CI**	**Deprivation gap (%)**	**95% CI**	**Deprivation gap (%)**	**95% CI**	**Deprivation gap (%)**	**95% CI**	**Deprivation gap (%)**	**95% CI**
1 year	Men	−2.9^**^	(−4.5, −1.2)	−5.1^**^	(−6.6, −3.6)	−7.1^**^	(−8.7, −5.6)	−2.2^**^	(−3.5, −1.0)	−7.8^**^	(−10.0, −5.6)
	Women	−4.2^**^	(−5.7, −2.6)	−5.3^**^	(−6.8, −3.9)	−6.7^**^	(−8.3, −5.1)	−1.4^*^	(−2.5, −0.2)	−6.8^**^	(−9.1, −4.5)
5 years	Men	−2.2^*^	(−4.1, −0.3)	−4.3^**^	(−6.1, −2.6)	−5.7^**^	(−8.0, −3.4)	−1.9^*^	(−3.4, −0.3)	−6.0^**^	(−8.8, −3.3)
	Women	−3.3^**^	(−5.0, −1.6)	−6.2^**^	(−7.8, −4.5)	−7.3^**^	(−9.4, −5.1)	−2.2^**^	(−3.6, −0.8)	−7.2^**^	(−9.8, −4.5)
10 years	Men	**−1.3**	(−3.5, 0.8)	−4.0^**^	(−6.3, −1.8)			**−2.7**	(−5.8, 0.4)	−5.3^**^	(−8.6, −2.1)
	Women	−3.1^**^	(−5.0, −1.3)	−7.1^**^	(−9.1, −5.1)			−4.0^**^	(−6.7, −1.3)	−7.9^**^	(−10.9, −4.9)

CI=confidence interval.

a Survival estimated with cohort or complete approach (see Rachet *et al*, 2008).

b Mean absolute change (%) in the deprivation gap in survival every 5 years, adjusted for the underlying trend in survival (see Rachet *et al*, 2008).

c Survival estimated with hybrid approach (see Rachet *et al*, 2008).

^*^P<0.05; ^**^P<0.01.
